# Inducible Cardiac-Specific Deletion of Sirt1 in Male Mice Reveals Progressive Cardiac Dysfunction and Sensitization of the Heart to Pressure Overload

**DOI:** 10.3390/ijms20205005

**Published:** 2019-10-10

**Authors:** Maria-Nieves Sanz, Lucile Grimbert, Maryline Moulin, Mélanie Gressette, Catherine Rucker-Martin, Christophe Lemaire, Mathias Mericskay, Vladimir Veksler, Renée Ventura-Clapier, Anne Garnier, Jérôme Piquereau

**Affiliations:** 1 UMR-S 1180, Inserm, Univ. Paris-Sud, Université Paris-Saclay, 92290 Châtenay-Malabry, France; maria.sanz@dbmr.unibe.ch (M.-N.S.); Lucile.grimbert@u-psud.fr (L.G.); maryline.moulin@univ-paris-diderot.fr (M.M.); melanie.gressette@u-psud.fr (M.G.); christophe.lemaire@u-psud.fr (C.L.); mathias.mericskay@inserm.fr (M.M.); vladimir.veksler@u-psud.fr (V.V.); Renee.ventura@u-psud.fr (R.V.-C.); anne.garnier@u-psud.fr (A.G.); 2 INSERM UMR_S 999, Hôpital Marie Lannelongue, 92350 Le Plessis Robinson, France; catherine.rucker-martin@u-psud.fr; 3 Université Versailles St. Quentin, Université Paris-Saclay, 78035 Versailles, France

**Keywords:** Sirtuin 1, heart, mitochondria, cardiac function

## Abstract

Heart failure is associated with profound alterations of energy metabolism thought to play a major role in the progression of this syndrome. SIRT1 is a metabolic sensor of cellular energy and exerts essential functions on energy metabolism, oxidative stress response, apoptosis, or aging. Importantly, SIRT1 deacetylates the peroxisome proliferator-activated receptor gamma co-activator 1α (PGC-1α), the master regulator of energy metabolism involved in mitochondrial biogenesis and fatty acid utilization. However, the exact role of SIRT1 in controlling cardiac energy metabolism is still incompletely understood and conflicting results have been obtained. We generated a cardio-specific inducible model of Sirt1 gene deletion in mice (*Sirt1^ciKO^*) to decipher the role of SIRT1 in control conditions and following cardiac stress induced by pressure overload. SIRT1 deficiency induced a progressive cardiac dysfunction, without overt alteration in mitochondrial content or properties. Sixteen weeks after *Sirt1* deletion an increase in mitochondrial reactive oxygen species (ROS) production and a higher rate of oxidative damage were observed, suggesting disruption of the ROS production/detoxification balance. Following pressure overload, cardiac dysfunction and alteration in mitochondrial properties were exacerbated in *Sirt1^ciKO^* mice. Overall the results demonstrate that SIRT1 plays a cardioprotective role on cardiac energy metabolism and thereby on cardiac function.

## 1. Introduction

Heart failure (HF), defined as the inability of the heart to provide adequate blood flow to meet the needs of the organism, is often the terminal end-point of different chronic cardiovascular diseases (CVD). Despite the advances of pharmaceutical therapeutic approaches over the past two decades, long term survival of HF patients still remains limited. This syndrome is thus a major cause of death worldwide and its prevalence is expected to rise in the ageing world population, prompting the research community to decipher the pathophysiology of HF. It has been demonstrated that HF is associated with profound modulations of energy metabolism of the heart; this altered energetics is thought to play major roles in the progression of the disease [1]. The failing heart is especially characterized by severe dysfunctions of the mitochondria, the main energy producers of the myocardium, and by an important decrease in fatty acid utilization in favor of carbohydrates at least at the beginning of the disorder. These alterations lead to an organ towards a state of depleted energy with lower concentrations of high-energy phosphate compounds (ATP, phosphocreatine) associated with elevated adenosine diphosphate (ADP) in the myocardium that is often described as an “engine out of fuel” [1]. Yet, this metabolic aspect of the disease is still not often taken into consideration by current standard HF therapies due to a lack of therapeutic targets.

The role of sirtuin 1 (SIRT1) in HF pathophysiology and its potential place in future therapies of this syndrome have aroused interest in recent years. This enzyme is a class III histone/protein deacetylase, whose activity depends on intracellular nicotinamide adenine dinucleotide (NAD^+^) level, presenting SIRT1 as a metabolic sensor of cellular energy [2] linking metabolic status of the cells to the regulation of gene expression. It is part of a family of highly conserved protein modifying enzymes firstly described in yeast. Mammals possess 7 sirtuins (SIRT1-7) and SIRT1 is the closest mammalian ortholog of silent mating type information regulation 2 (SIR2) described in yeast, drosophila melanogaster, and caenorhabditis elegans for its role in life extension in particular under caloric restriction [3,4,5]. SIRT1 deacetylates a large variety of substrates located in the nucleus or in the cytosol including histones, enzymes, transcriptional factors, and cofactors and consequently exerts essential functions in wide-ranging cellular processes such as energy metabolism, oxidative stress response, apoptosis, or aging [6]. It especially regulates energy metabolism by direct interaction with the peroxisome proliferator-activated receptor gamma co-activator 1α (PGC-1α), a master regulator of metabolism [7]. The SIRT1-mediated deacetylation of the latter results in the stimulation of its ability to co-activate a number of transcription factors controlling various facets of energy metabolism like mitochondrial biogenesis and fatty acid utilization [8,9]. The expression of PGC-1α has been shown to be largely reduced in various animal models of HF [10], as well as in cardiac tissue from HF human patients [11], especially in heart failure with reduced ejection fraction [12]. Knowing that a decrease in PGC-1α activity is associated with alterations of mitochondrial biogenesis and functions [13,14], stimulation of the SIRT1/PGC-1α axis could consequently be of great interest to restore cardiac energetics in a therapeutic context of CVD.

It has already been demonstrated that SIRT1 plays important roles in the maintenance of heart function and that it can be protective when the heart is subjected to stress like oxidative injury, hypertrophic stimuli, or ischemia/reperfusion injury (for review see [15]). In rodents, pharmacological activation of SIRT1 in the context of CVD can be beneficial [16,17]. This deacetylase could protect the heart through various mechanisms impacting processes like inflammation, fibrosis, apoptosis, energy metabolism, or calcium homeostasis [16,17,18]; however, the multitude of its targets renders the interpretation of cardiac protection by SIRT1 complex. In HF, the role of SIRT1 is still not perfectly understood. Its level is increased in the early stage of HF in animal models, especially in the nuclear fraction [19,20,21], while a clear reduction in SIRT1 protein content has been reported in advanced HF in rodents and humans [16,17,22,23,24]. Whereas a high SIRT1 content could be considered as an adaptive mechanism to face the increase in cardiac workload, studies have in contrast demonstrated that the increase in SIRT1 protein levels observed in the hypertrophic stage of pressure overload-induced myocardial dysfunction could be harmful [20,21]. Likewise, transgenic mouse models with constitutive high levels of SIRT1 overexpression develop cardiac dysfunctions associated with energy metabolism alterations [19,20,21,25]. Although the vast majority of the studies argue for the beneficial effect of SIRT1 in CVD, these contradictory results highlight the complexity of SIRT1 mechanism of actions in the cardiomyocyte and it seems necessary to clarify the roles of SIRT1 in the heart before considering this enzyme as a therapeutic target in such diseases, especially in HF.

Thus, the aim of the present study was to elucidate the role of SIRT1 under conditions of cardiac stress. For that, we used an original model of cardiac-specific knockout mice inducible in adult by tamoxifen injection.

## 2. Results

### 2.1. Cardiac Specific Tamoxifen-Induced Loss of Sirt1 in α-MHC-Cre/Flox Mice

**Figure 1:**
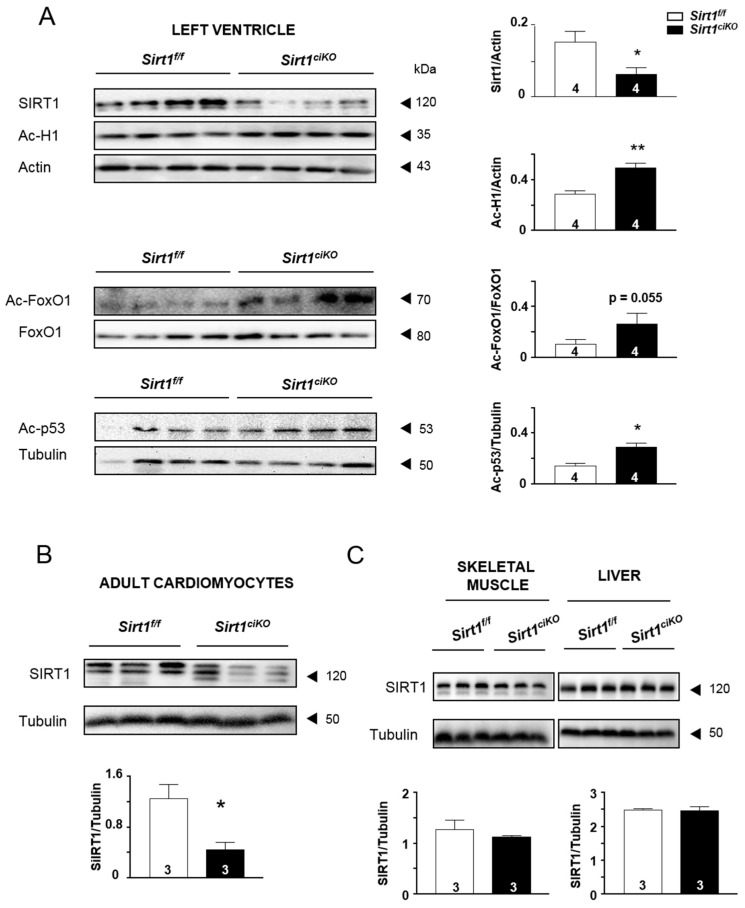
Cardiac-specific *Sirt1* inactivation in adult mice 4 weeks after tamoxifen injection. (**A**) Protein content of SIRT1 and its downstream acetylated targets (acetylated-Histone H1 (Ac-H1), acetylated-FoxO1 (Ac-FoxO1), and acetylated-p53 (Ac-p53) in left ventricle homogenates. (**B**) Protein content of SIRT1 in isolated cardiomyocytes. (**C**) Protein content of SIRT1 in skeletal muscle and liver homogenates. (*n* = 3 to 4 per experimental group), * *p* < 0.05; ** *p* < 0.01 *Sirt1^f/f^* versus *Sirt1^ciKO^*.

At 8 weeks of age, *Sirt1^ciKO^* mice were given tamoxifen to induce exon 4 excision from the floxed *Sirt1* alleles. Four weeks later, assessment of SIRT1 protein levels from LV homogenates revealed a reduction of 54 ± 11% in *Sirt1^ciKO^* mice in comparison with *Sirt1^f/f^* ones (Figure 1A). This was associated with a significantly higher acetylation level of histone H1 (H1) and tumor suppressor p53 protein (p53) as well as a strong trend towards an increase in acetylated forkhead box protein O1 (FoxO1) in *Sirt1^ciKO^* mice (*p* = 0.055). Inasmuch as the cardiomyocytes are not the only cell type encountered in the heart, the SIRT1 level was investigated in isolated cardiomyocytes to prove down-regulation of SIRT1 in these cells. Indeed, the SIRT1 level drastically dropped to 64 ± 8 % in cardiomyocytes isolated from *Sirt1^ciKO^* mice heart, although this protein did not completely disappear in this cell population (Figure 1B). Protein levels of SIRT1 in skeletal muscle and liver were similar in *Sirt1^ciKO^* and *Sirt1^f/f^* groups (Figure 1C). Altogether, the data confirmed the cardiac specificity of *Sirt1* deletion in the present animal model and a strong decrease in level/activity of this enzyme even though a small part of the cardiomyocyte population of *Sirt1^ciKO^* mice heart escapes the deletion process.

### 2.2. Sirt1^ciKO^ Mutants Develop a Mild Left Ventricular Systolic Dysfunction

**Figure 2:**
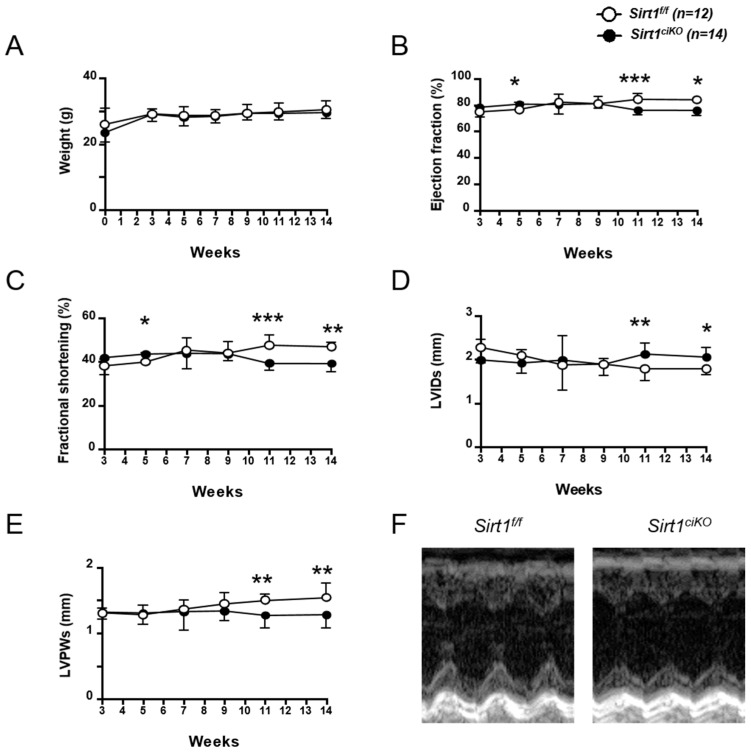
Progression of body weight and cardiac function of cardiac-specific knockout mice during 14 weeks after *Sirt1* deletion induction. (**A**) Body weight. (**B**) Left ventricular ejection fraction. (**C**) Left ventricular fractional shortening. (**D**) Left ventricular internal dimension at end-systole (LVIDs). (**E**) Left ventricular posterior wall thickness at end-systole (LVPWs). (**F**) Representative M-mode images of the left ventricle (14 weeks after induction of *Sirt1* deletion). (*n* = 12 to 14 per experimental group), * *p* < 0.05; ** *p* < 0.01; *** *p* < 0.001 *Sirt1^f/f^* versus *Sirt1^ciKO^*.

*Sirt1^ciKO^* mice body weight was similar to the controls during 14 weeks of observation (Figure 2A). Cardiac function of *Sirt1^ciKO^* mice and their control littermates were assessed by serial echocardiography 3, 5, 7, 9, 11, and 14 weeks after the first tamoxifen injection. While echocardiography parameters did not show any difference between control and mutant mice until 9 weeks after *Sirt1* deletion, significant decreases in LV ejection fraction (LVEF), fractional shortening (LVFS), and end-systolic left posterior wall thickness (LVPWs), as well as a significant increase in end-systolic left ventricular internal diameter (LVIDs) were observed 11 and 14 weeks after *Sirt1* deletion (Figure 2B–F and Table 1). However, these alterations of cardiac systolic function remained moderate after 14 weeks and no significant impact on cardiac output was noticed at this time point (Table 1). After 14 weeks, no difference in diastolic LV parameters was reported between *Sirt1^ciKO^* and *Sirt1^f/f^* mice (Table 1), and similar heart weight-to-body weight (HW/BW) and heart weight-to-tibia length (HW/TL) ratios in both groups indicated the absence of cardiac hypertrophy in *Sirt1^ciKO^* mice (Table 1).

Eleven months after tamoxifen injection, LV systolic parameters of *Sirt1^ciKO^* mice were much more altered. Clear decreases in LVEF and LVSF were observed and LVIDs was increased when mice were deleted for *Sirt1* (Table 1). These alterations were associated with an increase in LV end-systolic volume (ESV) and a decrease in cardiac output which is indicative of a cardiac dysfunction (Table 1). At this time point, the mutant mice had significantly lower weight than their littermates (Table 1). Despite alterations of cardiac function, heart weight, HW/BW, and HW/TL of these mice were not significantly different from control ones (Table 1). The comparable lung weight-to-tibia length (LW/TL) ratio between both mice groups suggests that *Sirt1^ciKO^* mice did not suffer from congestive heart failure yet (Table 1). These results indicate that this inducible cardiac-specific knockout mouse model developed a cardiac dysfunction with slow progression and, even though SIRT1 is known to regulate many cellular pathways, cardiac pump alterations were still mild 11 months after induction of *Sirt1* deletion.

### 2.3. Mitochondrial Oxidative Capacities are Preserved in Cardiac-Specific Sirt1 Mutant Mice

**Figure 3:**
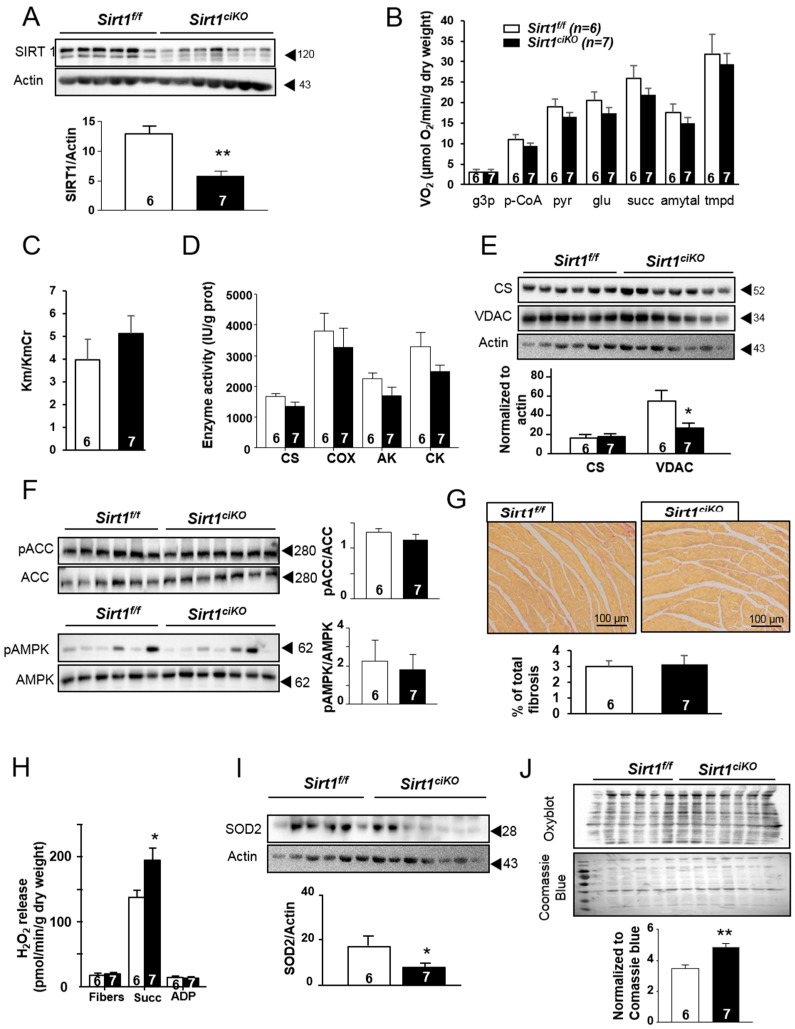
Cardiac mitochondrial phenotype of cardiac-specific knockout mice after 16 weeks of *Sirt1* deletion. (**A**) SIRT1 protein content in left ventricle (LV) homogenates. (**B**) Rate of respiration after successive addition of l-glycerol-3-phosphate (4 mM) (g3p), palmitoyl-CoA, and carnitine (100 µM and 2 mM) (p-CoA), pyruvate (1 mM) (pyr), glutamate (10 mM) (glu), succinate (15 mM) (succ), amytal (1 mM), and *N*, *N*′, *N*′-tetramethyl-phenylenediamine dihydrochloride (TMPD)-ascorbate (0.5 mM). (**C**) Ratio between Km without creatine and Km with creatine. (**D**) Citrate synthase (CS), cytochrome c oxidase (COX), adenylate kinase (AK), and creatine kinase (CK) enzymatic activities. (**E**) Immunoblotting of citrate synthase (CS) and voltage-dependent anion channel (VDAC) in LV homogenates. (**F**) Immunoblotting of total acetyl-CoA carboxylase (ACC), phosphorylated-ACC (pACC), total AMP-activated protein kinase (AMPK), and phosphorylated-AMPK (pAMPK) in LV homogenates. (**G**) Representative of fibrosis analysis by Sirius red staining of subequatorial heart sections. (**H**) Net rate of H_2_O_2_ release by the mitochondrial electron transport chain measured following sequential addition of succinate (5 mM) and ADP (1 mM). (**I**) SOD2 protein content in LV homogenates. (**J**) Carbonylation of LV proteins. (*n* = 6 to 7 per experimental group), * *p* < 0.05; ** *p* < 0.01 *Sirt1^f/f^* versus *Sirt1^ciKO^*.

Knowing the role of SIRT1 in cellular energetics and the high reliance of heart function on mitochondrial energy production, the consequences of *Sirt1* deletion on the cardiac mitochondria have been assessed. After 16 weeks of *Sirt1* deletion, while the SIRT1 level was drastically reduced (Figure 3A), mitochondrial respiration rates after cumulative addition of l-glycerol-3-phosphate, palmitoyl-CoA/carnitine, pyruvate, glutamate, succinate, amytal, and *N*, *N*′, *N*′-tetramethyl-phenylenediamine dihydrochloride (TMPD) were not significantly changed in *Sirt1^ciKO^* mice (Figure 3B), thereby indicating no impact of this deletion on mitochondrial substrate preferences and maximal mitochondrial oxidative capacities at this time point. Likewise, the comparable decrease by creatine in the Km ADP for respiration (Km/KmCr ratio) in both groups suggests that mitochondrial creatine kinase in *Sirt1^ciKO^* heart was fully functional (Figure 3C). This is associated with unchanged adenylate kinase (AK) and total creatine kinase (CK) enzymatic activity in both types of mice (Figure 3D), which suggests preserved energy transfers within the cardiomyocyte. The activity of citrate synthase (CS), traditionally used as a marker of functional mitochondrial mass, as well as cytochrome c oxidase (COX) activity were unchanged in mutant mice (Figure 3D). In accordance with these results, CS protein level was similar in both groups (Figure 3E). Although voltage-dependent anion channel (VDAC) protein levels showed a significant decrease after 16 weeks of *Sirt1* deletion (Figure 3E), the aforementioned results suggest that energy production function of cardiac mitochondria was not markedly altered in the mutants at this stage. A potential compensatory role of AMP-activated protein kinase (AMPK) in the maintenance of mitochondrial oxidative capacities in the *Sirt1^ciKO^* mice was not observed as phosphorylation of AMPK (Thr 172) and acetyl-CoA carboxylase (ACC) (Ser 79), used as markers of AMPK activity, were unchanged in comparison with *Sirt1^f/f^* (Figure 3F). Of note, no increase in total fibrosis was observed in mutant mice at this time point (Figure 3G).

The loss of *Sirt1* in the heart during 16 weeks was not without impact on mitochondrial functions since cardiac mitochondria of *Sirt1^ciKO^* mice released significantly more H_2_O_2_ than control mice when respiration was stimulated by succinate (Figure 3H). This was observed even though the MnSOD2 level was decreased in mutant mice (Figure 3I), indicating a higher propensity for mitochondria to produce superoxide anions during electron transfer within mitochondrial respiratory chain. In accordance with this increase in mitochondrial reactive oxygen species (ROS) production, assessment of protein carbonylation, a consequence of oxidative modifications of proteins by ROS, revealed a higher level of oxidative damage in mutants (Figure 3J), suggesting disruption of the balance between ROS production and detoxification systems.

**Figure 4:**
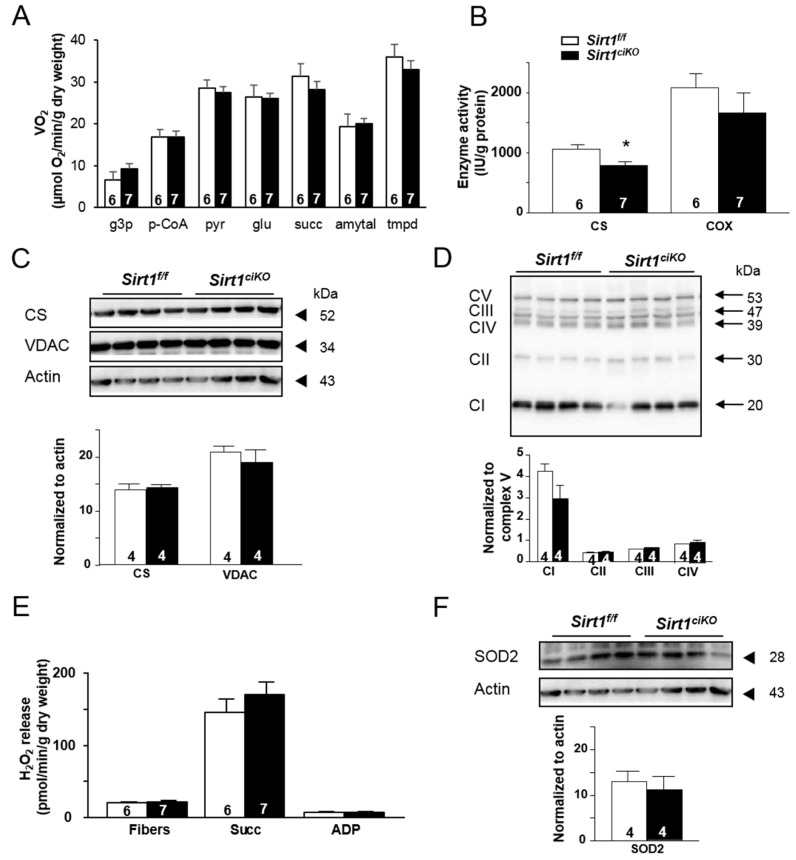
Cardiac mitochondrial phenotype of cardiac-specific knockout mice after 11 months of *Sirt1* deletion. (**A**) Rate of respiration after successive addition of l-glycerol-3-phosphate (4 mM) (g3p), palmitoyl-CoA and carnitine (100 µM and 2 mM) (p-CoA), pyruvate (1 mM) (pyr), glutamate (10 mM) (glu), succinate (15 mM) (succ), amytal (1 mM), and *N*, *N*′, *N*′-tetramethyl-phenylenediamine dihydrochloride (TMPD)-ascorbate (0.5 mM). (**B**) Citrate synthase (CS) and cytochrome c oxidase (COX) enzymatic activities. (**C**) Immunoblotting of citrate synthase (CS) and voltage-dependent anion channel (VDAC) in LV homogenates. (**D**) Total protein content of 5 subunits of oxidative phosphorylation complexes: C-I-20 (complex I (CI)), C-II-30 (complex II (CII)), C-III-Core 2 (complex III (CIII)), C-IV-COXI (complex IV (CIV)), and C-V-α (complex V (CV)). Protein content for CI, CII, CII, and CIV was normalized using CV as internal control. (**E**) Net rate of H_2_O_2_ release by the mitochondrial electron transport chain measured following sequential addition of succinate (5 mM), and ADP (1 mM). (**F**) SOD2 protein content in LV homogenates. (A–B and E: *n* = 6 to 7 per experimental group, C–D and F: *n* = 4 per experimental group), * *p* < 0.05 *Sirt1^f/f^* versus *Sirt1^ciKO^*.

Even eleven months after tamoxifen injection, when cardiac function alterations were much more pronounced, mitochondrial respiration still did not show any significant difference between *Sirt1^f/f^* and *Sirt1^ciKO^* mice (Figure 4A). The activity of CS was significantly reduced in *Sirt1^ciKO^* heart (Figure 4B) despite the fact that *Sirt1^ciKO^* mice exhibited mitochondrial oxidative capacities and CS protein content similar to control mice (Figure 4C). In contrast, COX activity was not impacted by *Sirt1* deletion as well as VDAC protein level (Figure 4C). No difference in mitochondrial electron transfer chain complexes between both groups of mice was evidenced by immunoblotting (Figure 4D). Whereas *Sirt1* deletion was associated with higher ROS production 16 weeks after tamoxifen injection (Figure 3H), this seemed to be normalized after 11 months since mitochondrial H_2_O_2_ release in presence of succinate was not significantly increased in the mutant mice (Figure 4E). Likewise, the MnSOD2 level was not reduced 11 months after induction of *Sirt1* deletion (Figure 4F).

**Figure 5:**
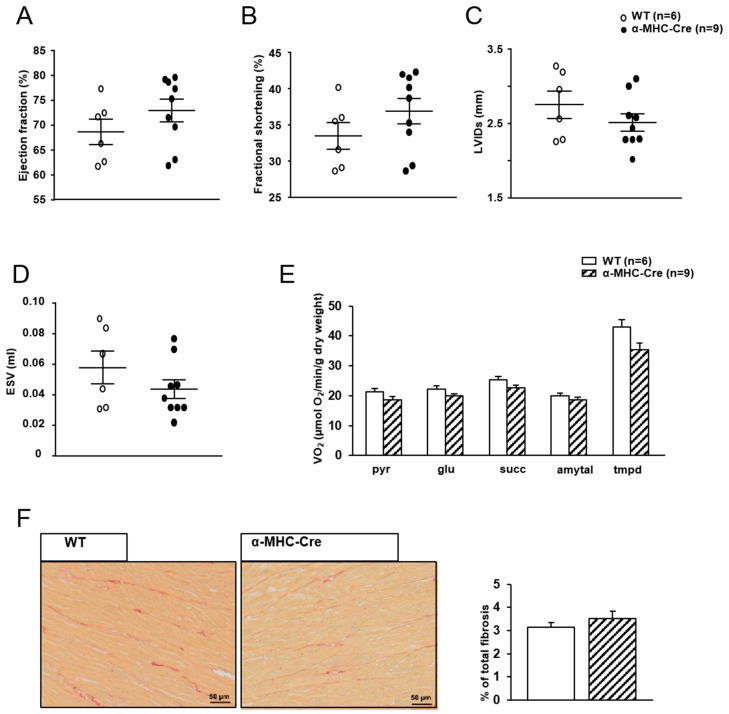
Cardiac phenotype of α-MHC-MerCreMer mice 16 weeks after tamoxifen injection. (**A**) Left ventricular ejection fraction. (**B**) Left ventricular fractional shortening. (**C**) Left ventricular internal dimension at end-systole (LVIDs). (**D**) Telesystolic LV volume (ESV). (**E**) Rate of respiration after successive addition of pyruvate (1 mM) (pyr), glutamate (10 mM) (glu), succinate (15 mM) (succ), amytal (1 mM), and TMPD-ascorbate (0.5 mM). (**F**) Representative pictures of fibrosis analysis by Sirius red staining of subequatorial heart section.

In order to confirm that cardiac alterations observed in this model were due to the loss of *Sirt1* and not to activation of the Cre-recombinase, the cardiac phenotype of α-MHC-MerCreMer mice treated with the same dose of tamoxifen (40 mg/kg i.p daily during 2 days) was investigated. Sixteen weeks after injection, cardiac functions were normal as judged by LVEF, LVFS, LVIDs, and ESV (Figure 5A–D). Mitochondrial oxidative capacities were not significantly different (Figure 5E) and no sign of fibrosis was evidenced by Sirius red staining (Figure 5F).

### 2.4. Sirt1^ciKO^ Mice Are More Sensitive to Cardiac Pressure Overload

**Figure 6:**
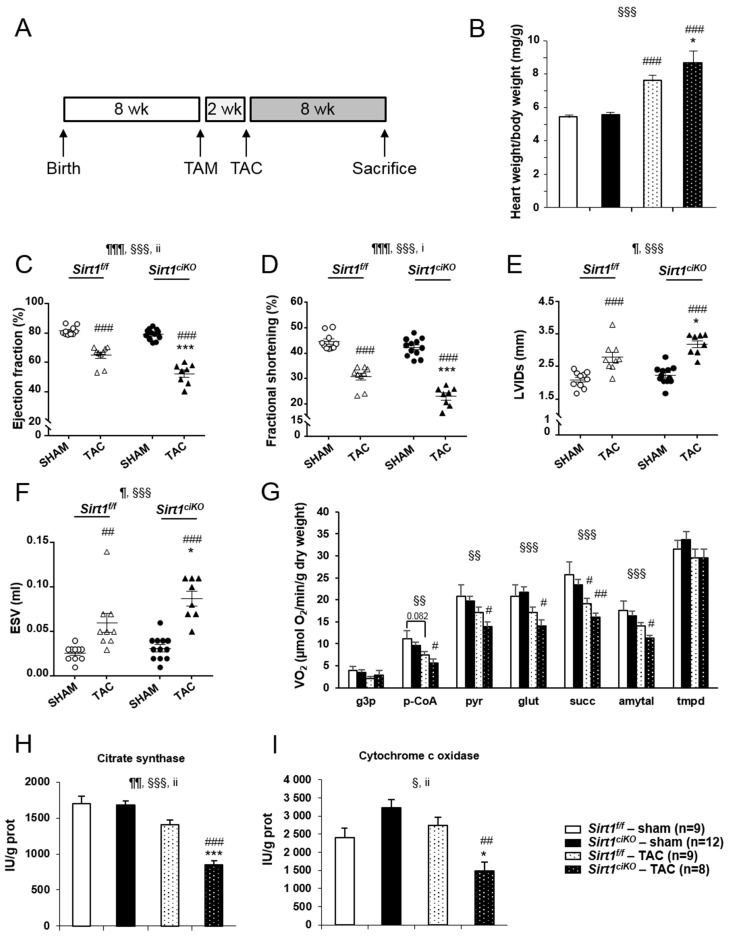
Cardiac phenotype of cardiac-specific knockout mice after 8 weeks of cardiac pressure overload. (**A**) Two weeks after tamoxifen injection, mice were subjected to surgery (TAC and sham) and were sacrificed 8 weeks later. (**B**) Heart weight-to-body weight ratio at sacrifice. (**C**) Left ventricular ejection fraction just before sacrifice. (**D**) Left ventricular fractional shortening just before sacrifice. (**E**) Left ventricular internal dimension at end-systole (LVIDs) just before sacrifice. (**F**) Telesystolic LV volume (ESV) just before sacrifice. (**G**) Rate of respiration after successive addition of l-glycerol-3-phosphate (4 mM) (g3p), palmitoyl-CoA and carnitine (100 µM and 2 mM) (p-CoA), pyruvate (1 mM) (pyr), glutamate (10 mM) (glu), succinate (15 mM) (succ), amytal (1 mM), and TMPD-ascorbate (0.5 mM). (**H**) Citrate synthase (CS) enzymatic activity. (**I**) Cytochrome c oxidase (COX) enzymatic activity. (*n* = 8 to 12 per experimental group), ANOVA: ¶ *p* ≤ 0.05, ¶¶ *p* ≤ 0.01, ¶¶¶ *p* ≤ 0.001 for the genotype effect; § *p* ≤ 0.05, §§ *p* ≤ 0.01, §§§ *p* ≤ 0.001 for the TAC effect; i *p* ≤ 0.05, ii *p* ≤ 0.01 for the interaction effect. Post hoc Newman–Keuls test: * *p* ≤ 0.05, ** *p* ≤ 0.01, *** *p* ≤ 0.001 *Sirt1^f/f^* versus *Sirt1^ciKO^* (same surgery); ^#^ *p* ≤ 0.05, ^##^ *p* ≤ 0.01, ^###^ *p* ≤ 0.001 sham versus TAC (same genotype).

**Figure 7:**
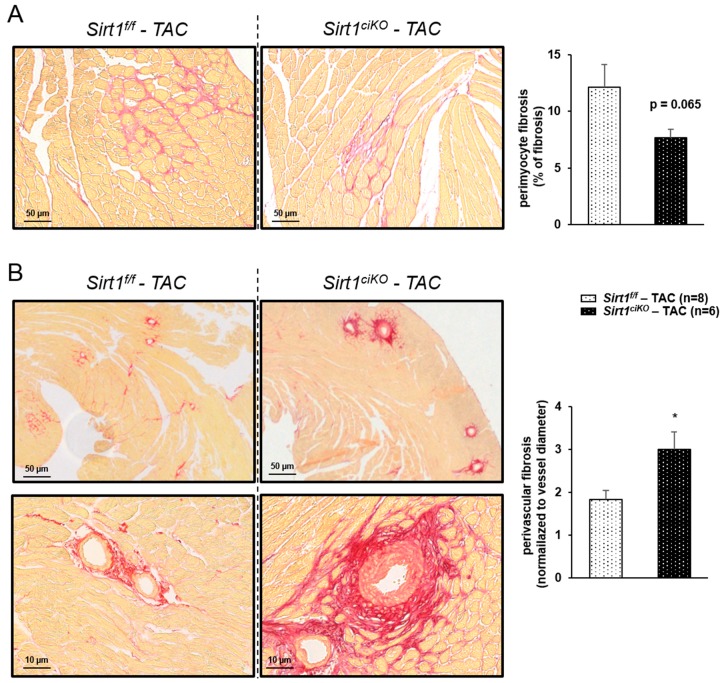
Myocardial fibrosis after 8 weeks of pressure overload. (**A**) Perimyocyte fibrosis analysis by Sirius red staining of subequatorial heart sections. (**B**) Perivascular fibrosis analysis by Sirius red staining of subequatorial heart sections. (*n* = 6 to 8 per experimental group), * *p* < 0.05 *Sirt1^f/^-TAC^f^* versus *Sirt1^ciKO^-TAC*.

Many studies have indicated that SIRT1 activation could be beneficial when the heart is subjected to various kinds of stress [7]. We thus investigated how cardiac-specific inducible *Sirt1* deletion triggered in adults impacted the response to TAC-induced cardiac pressure overload. To do so, mice underwent surgery 2 weeks after tamoxifen injection and were sacrificed 8 weeks later (Figure 6A). At sacrifice, mice of both TAC groups displayed an increase in heart weight compared to their respective sham group, as judged by absolute weight and HW/BW ratio (Table 2). However, the HW/BW ratio after TAC was much higher when mice did not express *Sirt1* (Table 2 and Figure 6B). No impact of pressure overload was observed on body and lung weights in any group while TAC induced a decrease in kidney weight independently of the genotype (Table 2). Echocardiography parameters determined just before sacrifice indicate that the decreases in left ventricular ejection fraction (LVEF) and fractional shortening (LVFS) after 8 weeks of TAC were exacerbated by *Sirt1* deletion (Table 2 and Figure 6C,D). The clear increases in LVIDs and ESV observed in both TAC groups were also significantly higher in *Sirt1^ciKO^* mice (Table 2 and Figure 6E,F). Mitochondrial respiration rates after addition of p-CoA, pyruvate, glutamate succinate, and amytal were reduced by pressure overload (Figure 6G). However, only *Sirt1^ciKO^* mice group showed statistically significant decrease in all these parameters when compared with respective sham values. Respiration rates measured from permeabilized cardiac fibers prepared from TAC *Sirt1^f/f^* mice exhibited a lower mean value than sham *Sirt1^f/f^* mice after the addition of these substrates but a significant difference between these groups was only observed after succinate addition (Figure 6G). Although not significant, respiration rates displayed the same trend towards lower values in TAC *Sirt1^ciKO^* group in comparison with TAC *Sirt1^f/f^* mice. Analysis of mitochondrial respiration assay combined with large drops of CS and COX activities only in TAC mutant *Sirt1^ciKO^* mice (Figure 6H,I) strongly suggests that, in the context of cardiac pressure overload, mitochondrial oxidative capacities were more severely altered when *Sirt1* was deleted in cardiomyocytes. Pressure overload is also associated with fibrosis in *Sirt1^f/f^* and *Sirt1^ciKO^* mice as revealed by Sirius red staining. Perimyocyte interstitial fibrosis was not significantly different in TAC *Sirt1^ciKO^* mice compared to TAC controls (Figure 7A), but perivascular fibrosis was significantly more marked in this group than in controls (Figure 7B).

## 3. Discussion

Despite the extensive study of SIRT1 and its role in the heart over the past twenty years, the consequences of a cardiomyocyte-specific and inducible loss of *Sirt1* at adult stage have been poorly addressed. We thus generated a murine model with a truncation of a part of the catalytic domain of *Sirt1* (Exon 4) specifically in cardiomyocytes. At baseline, the specific loss of SIRT1 in cardiomyocytes of young adult mice leads to a slight and progressive drop in the left ventricular systolic function. Sixteen weeks after deletion, this cardiac dysfunction was associated with a higher mitochondrial ROS production and marked oxidative damage whereas no alteration in mitochondrial oxidative capacities was noticed. Strikingly, this left ventricular systolic dysfunction gets worse in old KO mice (11 months after tamoxifen injection) even though the mitochondrial propensity to produce more ROS and protein oxidative alterations observed after 16 weeks were not found in these elderly *Sirt1* deficient mice. Beyond these results obtained in young and aged mice at basal conditions, we show that the specific loss of *Sirt1* in adults exacerbates the cardiac dysfunction induced by pressure overload. This dysfunction is accompanied by more pronounced mitochondrial alterations as well as a more severe perivascular fibrosis.

*Sirt1* deletion was induced when mice reached an adult and mature state. This model is attractive since it has been shown that the total and constitutive deficiency of SIRT1 is associated with a very high perinatal mortality and a severe dilated cardiomyopathy when animals survive and reach adult age [26]. Unlike the constitutive deficient models, it makes possible to investigate the consequences of a late loss of functional SIRT1 on heart function and to study the development and progression of HF in the absence of SIRT1. To generate the present specific transgenic mouse line, mice carrying the α-MHC-MerCreMer transgene were used, thereby allowing the expression of a tamoxifen-inducible Cre-recombinase under the control of the mouse alpha-myosin heavy chain promoter (only expressed in the cardiomyocytes of heart). Of note, several studies have reported a cardiac toxicity of the Cre-recombinase depending on the duration and the level of Cre expression [27,28,29]. Thus, mice carrying α-MHC-MerCreMer transgene are likely to develop cardiac dysfunction associated with fibrosis, cell infiltration, and inflammation when treated with repetitive doses of tamoxifen [27,30]. Nevertheless, Lewox and collaborators have demonstrated that a single tamoxifen injection (40 mg/kg) successfully induces robust α-MHC-MerCreMer-dependent recombination without exhibiting any cardiotoxicity. Based on this study and preliminary tests led in our lab (data not shown), a 40 mg/kg/day tamoxifen intraperitoneal injection for 2 consecutive days has been chosen as standard protocol to induce Cre-recombinase activation in adult α-MHC-Cre/flox mice. The total absence of cardiac dysfunction and fibrosis in α-MHC-MerCreMer mice treated with this same dose of tamoxifen confirmed that the alterations observed in the present study were due to the loss of *Sirt1* and not to an excessive activation of the Cre-recombinase.

In KO mice under basal conditions, the modest alterations of several cardiac contractile parameters (LVEF, LVFS, IVSs, LVIDs, and LVPWs) suggest a mild ventricular systolic dysfunction. This degradation of cardiac function appears 11 weeks after tamoxifen injection, becomes more important after 11 months of *Sirt1* deletion and was not associated with major anatomical changes when compared to control mice. Importantly, the echocardiography analysis revealed that diastolic function is normal and in particular no significant dilatation has been observed during the relaxation of the left ventricle when *Sirt1^ciKO^* mice are compared to *Sirt1^f/f^*. Interestingly, mice displaying a non-tissue specific complete (homozygous) or partial (heterozygous) constitutive SIRT1 deficiency exhibit a dilated cardiomyopathy at 5 months of age which is not associated with cardiomyocyte hypertrophy [26]; the cross-sectional area of cardiomyocytes was even reduced in this model.

Surprisingly, given that SIRT1 has been extensively studied for its role in energy metabolism [6], the specific cardiac inducible deletion of *Sirt1* does not provoke major mitochondrial dysfunction even after 11 months of deletion. This could be explained by the fact that SIRT1 is an enzyme activated by an increase in NAD^+^ level that is a hallmark of energetic stress. Under standard conditions and in absence of stress, the consequence of the loss of SIRT1 functions could thus be modest, practically imperceptible, and too mild to be evidenced when investigating mitochondrial functions. Moreover, even though the deletion is induced at adult stage, chronic compensatory mechanisms cannot be excluded and could progressively be established, explaining for instance the normalization of mitochondrial ROS production in old mice whereas mitochondria significantly produce more ROS after 16 weeks of *Sirt1* deletion. Of note, this increase in ROS production in absence of *Sirt1* expression was expected given the role of this enzyme in antioxidant defenses, in particular through FoxOs activation [7]. On the other hand, the mechanism allowing the normalization of these parameters in old mice has not been identified in this study though we could eliminate the hypothesis a compensatory activation of AMPK. The requirement of SIRT1 for AMPK activation via liver kinase B1 (LKB1) deacetylation could explain this absence of AMPK stimulation [31].

In the aforementioned study using constitutive *Sirt1* KO mice, cardiac alterations were associated with important mitochondrial alterations such as aberrant mitochondrial structure, lesser mitochondrial content, or reduced mitochondrial-DNA encoded genes [26], suggesting that the myocardium is more damaged in this model. The differences with the present study could be attributed to peripheral disorders caused by a total loss of SIRT1 in other cell types inducing for example a vascular dysfunction that could affect the heart [32,33] or to the roles of SIRT1 in the prenatal and postnatal development of the heart and cardiomyocyte differentiation [34,35,36], thereby inducing mitochondrial defects that will exacerbate cardiac dysfunction. The cardiac specific non-inducible *Sirt1* KO murine model developed by Hsu and collaborators in which the expression of the Cre-recombinase is under the control of α-myosin heavy chain promoter (αMHC-Cre) is in line with these hypotheses [37]. Indeed, these cardiac specific KO *Sirt1* mice exhibit a normal cardiac phenotype at 3 months of age. This could be explained not only by the fact that *Sirt1* ablation is restricted to the heart but also by the low induction of α-MHC expression during the fetal life and early postnatal development in rodents [38]. Therefore, SIRT1 protein level and activity could be sufficiently maintained during prenatal development, thereby giving newborns with normal heart. The expression of *Sirt1* would then progressively decline during postnatal development, a period during which α-MHC-Cre expression progressively increases [38,39]. Everything goes as if *Sirt1* deletion was progressively induced in the days following birth in this model and the normal cardiac function displayed by these mice at 3 months of age could be due to the slowness of the development of cardiac dysfunction after *Sirt1* loss. This is in accordance with the absence of cardiac dysfunction during several weeks after tamoxifen injection in our model. Incidentally, this non-inducible cardiac-specific *Sirt1* deletion under α-MHC promoter control makes the mice more sensitive to stress and leads to an increase in oxidative damage in 6-month old mice and to a progressive impaired left ventricle contractility that becomes significant in 12-month old mice [37,40]. This sequence marked by an oxidative stress followed by a more severe degradation of cardiac function in old mice strongly evokes what is observed in our inducible cardiac-specific *Sirt1* knockout model, highlighting some similarities between both animal models.

Given the important role of SIRT1 in aging [19,40], particular attention was paid to the inducible cardiac-specific *Sirt1* knockout mice at an advanced age. Eleven months after tamoxifen injection, the systolic dysfunction is worsened, as revealed by greater LVIDs and reduced IVSs in comparison with younger mice (14 weeks after tamoxifen injection). Interestingly and as it has been reported in humans [41], old mice show incipient diastolic dysfunction (as shown by higher LVIDd); however, it is not affected by the loss of SIRT1. The slow onset of systolic dysfunction in this model raises questions that will need further investigations. Indeed, although a very slight dysfunction is observed after 11 weeks of *Sirt1* ablation, one can wonder to what extent the significant alterations reported in 11-month old mice are the result of small and early cellular homeostasis modifications that slowly impact cardiac function as time goes by, or if they are the consequences of the impairment of phenomena requiring SIRT1 during aging, such as the regulation of endoplasmic reticulum (ER) stress induced by aging [40]. The fact that cardiac dysfunction in elderly mice is not associated with clear degradation in mitochondrial respiration and/or higher oxidative stress is quite unexpected since SIRT1 has largely been described as an important actor of cellular energetics and antioxidant defenses [6]. Nevertheless, the noticeable decrease in CS activity in these old *Sirt1^ciKO^* mice suggest perturbations of energy metabolism even though they are not revealed by the assessment of maximal mitochondrial oxidative capacities in permeabilized fibers or mitochondrial protein content. In the light of this study, it seems that alterations of the mitochondria as the main energy producer of the cardiomyocyte would not be the major cause of the cardiac dysfunction following the cardiac-specific deletion of *Sirt1* in adults. Additional analysis is required to better understand the profound modulations of energy metabolism in this *Sirt1^ciKO^* mice and to determine if other processes could be involved in the establishment of the systolic dysfunction. For instance, SIRT1 is known to be involved in regulation of apoptosis, autophagy, calcium homeostasis, or ER stress [16,42,43], processes that have to be tightly balanced in the healthy heart. Indeed, using a constitutive model of *Sirt1* deletion, we have shown recently that SIRT1 is a novel regulatory mechanism for protecting cardiac cells from ER stress [44]. Thus, the potential dysregulation of these processes in this inducible cardiac-specific *Sirt1* KO model will be the subject of further studies.

Different models of *Sirt1* deficient mice have been described in the past few years and they all displayed an increased sensitivity to cardiac stress when the expression of this protein is reduced or abolished (for review see [7]). Again, in this inducible cardiac-specific *Sirt1* KO model, the loss of SIRT1 function exacerbates cardiac dysfunction induced by pressure overload. This result reinforces the idea that SIRT1 plays a protective role when the heart is subjected to a stress. Although a few studies have created doubt regarding the cardioprotective effect of SIRT1 [20,21], the observations made in the present study are perfectly in line with numerous studies showing that SIRT1 is beneficial in the heart facing various stresses such as oxidative stress or ischemia/reperfusion [19,31,37]. As already shown [45], TAC-induced cardiac dysfunction is associated with decreases in mitochondrial respiration rates stimulated by different substrates; these alterations tend to be more pronounced in the absence of SIRT1. The exacerbation of CS and COX activity drop in *Sirt1^ciKO^* subjected to TAC clearly demonstrates that SIRT1 is required to maintain cellular energetics under stress conditions. However, although the energy metabolism alterations induced by TAC are more severe in *Sirt1^ciKO^* mice, mitochondrial oxidative capacities are not drastically reduced when compared to TAC *Sirt1^f/f^* mice, suggesting that the much higher sensitivity to cardiac pressure overload of these mutant mice might be multifactorial.

For instance, it clearly appears that TAC induces fibrosis. It is well known that fibrosis changes tissue properties and negatively affects heart function. Although the loss of *Sirt1* was not associated with fibrosis under basal conditions or with higher perimyocyte fibrosis after TAC, the fact that *Sirt1^ciKO^* mice heart subjected to TAC developed more perivascular fibrosis could be in part responsible for the hypersensitivity of these mice to pressure overload. The link between SIRT1 and fibrosis has already been studied and it has especially been shown that SIRT1 regulates fibrosis though the TGF-β1/Smad2/3 axis and stimulation of SIRT1 by SRT1720 reduces cardiac fibrosis and improves cardiac function [17]; this could thus be one of the facets of the protective role of SIRT1. In the present genetically modified mice, the specific increase in perivascular fibrosis was unexpected and raises questions. Indeed, concern still persists about how the deletion of *Sirt1* in cardiomyocytes results in the higher collagen deposition specifically in the vicinity of the vessels when the heart is stressed. The answer may have to be sought on the side of the impairment of vessel permeability due to some factors released by cardiomyocytes or circulating factors. Indeed, this perivascular fibrosis reminds what was observed and described by Heath and Edwards [46] in pulmonary arterial hypertension in which perivascular fibrosis is preceded by a permeability loss-induced edema. This will need further studies to confirm this mechanism and decipher the role of SIRT1 in the complex interplay between the different cell types of the heart.

Our study also shows that cardiac hypertrophy in response to pressure overload is possible in the absence of SIRT1 even though the hypertrophic role of SIRT1 has been documented [18,20,26,47]. This points out the intricate role of SIRT1 in a process in which it has sometimes been described as a pro-hypertrophic or anti-hypertrophic factor [17,48,49]. This paradoxical effect of SIRT1 in a given process highlights the complexity of its functions that may be modulated according to its activation level. The generation of several models of SIRT1 overexpression illustrates this remark since the phenotype is intimately dependent on the level of SIRT1 [25], thereby highlighting that this deacetylase could act in a dose-effect manner.

Cardiac-specific inducible *Sirt1* deletion model proved helpful in deciphering the role of Sirt1 in basal and TAC conditions. Although *Sirt1* deletion induced a progressive cardiac dysfunction, this was observed without overt mitochondrial dysfunction. However, the role of SIRT1 in mitochondrial and cardiac function defects was evidenced following pressure overload induced by aortic constriction showing the importance of this pathway in cardioprotection.

## 4. Materials and Methods

### 4.1. Animals

*Sirt1^floxΔE4/floxΔE4^* (*Sirt1^f/f^*) homozygous mice (kindly provided by David A Sinclair’s group) [36] and heterozygous Myh6-MerCreMer^+/−^ (*α-MHC-Cre*) mice [50] were crossed to create cardiac-specific and inducible knock-out (*Sirt1^ciKO^*) mice (*α-MHC-Cre*/*Sirt1^f/f^*) using Cre-lox technology [51]. Briefly, exon 4 of *Sirt1* gene was flanked with two LoxP sites that were recognized and excised by tamoxifen activated Cre recombinase. The cardiac specificity of the deletion was ensured by the fact that the expression of Cre recombinase was under the control of α-MHC. Male *Sirt1^ciKO^* mice were injected with tamoxifen (40 mg/kg i.p daily during 2 days) at the age of 8 weeks to induce *Sirt1* deletion, thereby generating *Sirt1* cardiac-specific inducible mice called *Sirt1^ciKO^* in this study. Littermate *Sirt1^f/f^* mice not carrying *α-MHC-MerCreMer* transgene were subjected to the same tamoxifen treatment and were used as control mice. While some of these mice were sacrificed 16 weeks or 11 months after tamoxifen injection with no intervention other than the latter injection, others underwent surgery to induce pressure overload by transverse aortic constriction (TAC) 2 weeks after injection. Anaesthesia was induced by intraperitoneal injection of ketamine (50 mg/kg) and xylazine (8 mg/kg) and silk suture were placed around the aorta using a blunted 27-gauge needle to generate aortic stenosis. Animals were euthanized 8 weeks after surgery by cervical dislocation and hearts were rapidly excised, rinsed in cold calcium-free Krebs solution and weighed. A part of the left ventricle (LV) was immediately used for mitochondrial function assessment and another part was flash frozen in liquid nitrogen for further biochemical determinations. All animal experimental procedures were approved by animal ethics committee of Paris-Sud University, authorized by French government (authorization number: B9201901, APAFIS#2317-2015100615037480 (approved on November 3^rd^, 2015)) and complied with directive 2010/63/EU of the European Parliament on the protection of animals used for scientific purposes. 

### 4.2. Echocardiography

Echocardiography was done using a 12 MHz transducer (Vivid 7, General Electric Healthcare, General Electric Healthcare, Chicago, IL, USA) under 2.5% isoflurane gas anaesthesia to assess cardiac function. M-mode echocardiography was used to determine left ventricular mass, fractional shortening, and left ventricular ejection fraction.

### 4.3. Ventricular Cardiomyocyte Isolation

Immediately after sacrifice, the heart from *Sirt1^f/f^* or *Sirt1^ciKO^* mice four weeks after tamoxifen injection was washed and aorta cannulated in washing buffer (in mM: 113 NaCl, 4.7 KCl, 1.2 MgSO_4_, 0.6 KH_2_PO_4_, 0.6 NaH_2_PO_4_, 10 HEPES, 1.6 NaHCO_3_, 30 Taurine, 20 Glucose) as previously described [52]. The heart was then retrogradely perfused at a constant hydrostatic pressure with digestion buffer (washing buffer added with 80 µg/mL Liberase™ research grade (Roche)) for 6–9 min at 37 °C. The ventricles of the digested heart were excised, shredded into small pieces in the collecting buffer (washing buffer added with 0.2 mM CaCl_2_ and 5 mg/mL BSA), and gently mechanically shaken to release the cardiomyocytes. After filtration and 10 minutes of decantation, only the cardiomyocyte pellet was kept and resuspended in selecting buffer 1 (washing buffer added with 0.5 mM CaCl_2_ and 5 mg/mL BSA). After a 10 min period of decantation, a new resuspension/decantation step was carried out in selecting buffer 2 (washing buffer added with 1 mM CaCl_2_). Finally, the cardiomyocytes pellet obtained was frozen in liquid nitrogen for further analysis.

### 4.4. Mitochondrial Functional Assays in Permeabilized Cardiac Fibers

Fibers prepared from the left ventricle were permeabilized with saponin as previously described [53] and kept on ice until use in S buffer (in mM: 2.77 CaK_2_ ethyleneglycol tetraacetic acid (EGTA), 7.23 K_2_EGTA (100 nM free Ca^2+^), 6.56 MgCl_2_ (1 mM free Mg^2+^), 5.7 Na_2_ATP, 15 phosphocreatine, 20 taurine, 0.5 dithiothreitol (DTT), 50 K-methane sulfonate (160 mM ionic strength), 20 imidazole, pH 7.1). Measurements aimed at determining mitochondrial parameters were expressed per gram of dry fiber weight.

### 4.5. Mitochondrial Respiration

Mitochondrial respiratory function was studied in situ in saponin-permeabilized cardiac muscle fibers using a Clarke electrode as previously described (Kuznetsov 2008). A protocol was designed to measure oxygen consumption after successive addition of ADP (2 mM), malate (4 mM), l-glycerol-3-phosphate (4 mM), palmitoyl-CoA and carnitine (100 µM and 2 mM), pyruvate (1 mM), glutamate (10 mM), succinate (15 mM), amytal (an inhibitor of complex I, 1 mM), and the complex IV substrates *N*, *N*′, *N*′-tetramethyl-phenylenediamine dihydrochloride (TMPD)-ascorbate (0.5 mM) (activator of complex IV) to respiration solution (in mM: 2.77 CaK_2_ ethyleneglycol tetraacetic acid (EGTA), 7.23 K_2_EGTA (100 nM free Ca^2+^), 1.38 MgCl_2_, 3 K_2_HPO_4_, 20 taurine, 0.5 dithiothreitol (DTT), 90 K-methane sulfonate and 10 Na-methane sulfonate, 20 imidazole, pH 7.1) at 23 °C. Rates of respiration are given in µmoles O_2_/min/g dry weight. A second protocol was designed to determine ADP sensitivity of mitochondria and functioning of mitochondrial creatine kinase. For that, respiration was stimulated by the addition of 100 µM ADP in the presence of glutamate (10 mM) and malate (4 mM). Creatine (11 mM) was then added to the chamber and finally the maximal respiration rate (Vmax) was measured by adding 2 mM ADP. The apparent Km values in the presence and absence of creatine were calculated as previously described [39].

### 4.6. Mitochondrial H_2_O_2_ Release

H_2_O_2_ released by respiring mitochondria was determined in permeabilized cardiac fibers as previously described [54]. Before measurement, fibers were removed from S buffer and washed in Z1 buffer (in mM: 35 KCl, 1 EGTA, 3 MgCl_2_, 10 K_2_HPO_4_, 10 K-MES, 0.5 mg/mL BSA, pH 7.3 at 4 °C) on ice. Fibers were then incubated in Z2 buffer (in mM: 35 KCl, 1 EGTA, 3 MgCl_2_, 10 K_2_HPO_4_, 10 K-MES, 0.5 mg/mL BSA, pH 7.3 at 37 °C) containing horseradish peroxidase (1.2 U/mL) and Amplex red (20 µM: excitation-emission: 563 to 587 nm) at 37 °C in a fluorescence spectrophotometer (F-2710, Hitachi) under gentle agitation. Baseline fluorescence was measured in the absence of any exogenous respiratory substrates before sequential addition of succinate (5 mM) and ADP (1 mM). Rates of H_2_O_2_ production were calculated using a standard curve established under the same experimental conditions.

### 4.7. Enzyme Activity

Frozen tissue samples were weighed, homogenized (Bertin Precellys 24) in ice-cold buffer (50 mg/mL) containing 4-(2-hydroxyethyl)-1-piperazineethanesulfonic acid (HEPES) 5 mM (pH 8.7), EGTA 1 mM, DTT 1 mM, and 0.1% Triton X-100. Activity of citrate synthase (CS) and cytochrome c oxidase (COX) was determined using standard spectrophotometric assays [55,56].

### 4.8. Immunoblotting

Frozen tissue samples were homogenized (Bertin Precellys 24) in ice cold buffer containing HEPES 50 mM, KCl 50 mM, ethylenediaminetetraacetic acid (EDTA) 1 mM, β-glycerophosphate 5 mM, Triton X-100 0.1%, orthovanadate 1 mM, dithithreitol 1 mM, sodium fluoride 50 mM, Na pyrophosphate 5 mM, phenylmethylsulfonyl fluoride 0.2 mM, and antiprotease cocktail set (Calbiochem 539134). Protein extracts were separated on SDS-polyacrylamide gel (8% to 12%) and then transferred to polyvinylidene difluoride membranes for Western blot. After 1 hour of blocking in PBS containing TWEEN20 (0.1%) and non-fat milk (5%), the membranes were incubated overnight at 4 °C with primary antibody (Table 3). After washing, the membranes were incubated with a secondary antibody coupled with horseradish peroxidase for 1 hour at room temperature and visualized using chemiluminescent substrate (Luminata™ Western Chemiluminescent HRP Substrates, Millipore). Light emission was detected by autoradiography and quantified using an image-analysis system (iBright FL1000, Invitrogen, Waltham, MA, USA).

### 4.9. Histological Analysis

Hearts were fixed in 4% paraformaldehyde, paraffin embedded and serially sectioned (5 μm). Sections were stained with Sirius red. Fibrosis quantification was performed on 3–4 sections (5–10 fields/section) per animal using Image J software.

### 4.10. Statistical Analysis

For TAC induced-pressure overload, *Sirt1^f/f^* or *Sirt1^ciKO^* mice were randomly assigned to SHAM and TAC group. All results are expressed as mean ± SEM. Data were analyzed using Statistica software (Statistica, Statsoft Inc., Tulsa, OK, USA). To assess significance, we performed Student’s t test when comparing only 2 groups or two-way ANOVA for independent factors when appropriate for the experimental design followed by Newman–Keuls post-hoc tests to identify significant differences between means. Differences between groups were considered significant if *p*-value was <0.05.

## Figures and Tables

**Table 1:** Anatomical and echocardiographic parameters 14 weeks and 11 months after induction of *Sirt1* deletion by tamoxifen injection. BW, Body weight. TL, Tibia length. HW, Heart weight. HW/BW, Heart weight-to-body weight ratio. HW/TL, Heart weight-to-tibia length ratio. LW/BW, Lung weight-to-body weight ratio. LW/TL, Lung weight-to-tibia length ratio. KW/BW, Kidney weight-to-body weight ratio. KW/TL, Kidney weight-to-tibia length ratio. HR, Heart rate. IVSd, Interventricular septal thickness at end-diastole. IVSs, Interventricular septal thickness at end-systole. LVIDd, Left ventricular internal dimension at end-diastole. LVIDs, Left ventricular internal dimension at end-systole. LVWPd, Left ventricular posterior wall thickness at end-diastole. LVWPs, Left ventricular posterior wall thickness at end-systole. EDV, Left ventricular telediastolic volume. ESV, Left ventricular telesystolic volume. LVEF, Left ventricular ejection fraction. LVFS, Left ventricular fractional shortening. SV, Stroke volume. LV, left ventricular mass. CO, Cardiac output. ANOVA: ¶ *p* ≤ 0.05, ¶¶ *p* ≤ 0.01, ¶¶¶ *p* ≤ 0.001 for the genotype effect; § *p* ≤ 0.05, §§ *p* ≤ 0.01, §§§ *p* ≤ 0.001 for the aging effect; i *p* ≤ 0.05, ii *p* ≤ 0.01 for the interaction effect. Post hoc Newman–Keuls test: * *p* ≤ 0.05, ** *p* ≤ 0.01, *** *p* ≤ 0.001 *Sirt1^f/f^* versus *Sirt1^ciKO^* (same age); ^$^ *p* ≤ 0.05, ^$$^ *p* ≤ 0.01, ^$$$^ *p* ≤ 0.001 young versus old (same genotype).

	14 Weeks		11 Months		2-Way ANOVA
	*Sirt1^f/f^*	*Sirt1^ciKO^*	*Sirt1^f/f^*	*Sirt1^ciKO^*	
BW (g)	30.4 ± 0.4	29.9 ± 0.3	33.4 ± 1.5 ^$$^	29.6 ± 0.6 **	¶¶, i
TL (mm)	17.2 ±0.2	17.2 ± 0.1	18.8 ± 0.3 ^$$^	18.6 ± 0.7 ^$^	§§§
HW (mg)	143.2 ± 4.4	144.8 ± 3.1	173.6 ± 3.9 ^$$^	162.6 ± 10.4 ^$^	§§§
HW/BW (mg/g)	4.7 ± 0.1	4.8 ± 0.1	5.2 ± 0.2	5.5 ± 0.3 ^$^	§§
HW/TL (mg/mm)	8.3 ± 0.2	8.6 ± 0.1	9.2 ± 0.2	8.8 ± 0.2	
LW/BW (mg/g)	4.5 ± 0.1	4.9 ± 0.2	5.2 ± 0.2 ^$$^	5.3 ± 0.1	§§§
LW/TL (mg/mm)	8.1 ± 0.2	8.6 ± 0.3	9.3 ± 0.5	8.7 ± 0.4	/
KW/BW (mg/g)	11.8 ± 0.2	12.1 ± 0.3	12.6 ± 0.9	12.2 ± 0.6	/
KW/TL (mg/mm)	20.9 ± 0.4	21.5 ± 0.5	22.2 ± 0.9	18.7 ± 1.2 ^$,^*	i
HR (bpm)	506 ± 7	498 ± 11	542 ± 13	549 ± 16	§§
IVSd (mm)	1.04 ± 0.04	0.93 ± 0.03	0.82 ± 0.12	0.82 ± 0.07	§§
IVSs (mm)	1.64 ± 0.03	1.44 ± 0.03 **	1.44 ± 0.06 ^$$^	1.28 ± 0.03 ^$,^*	¶¶¶, §§§
LVIDd (mm)	3.36 ± 0.06	3.39 ± 0.06	3.82 ± 0.15 ^$^	3.81 ± 0.13 ^$$^	§§§
LVIDs (mm)	1.78 ± 0.04	2.06 ± 0.06 *	2.09 ± 0.12^$^	2.58 ± 0.12 ^$$$,^***	¶¶¶, §§§
LVPWd (mm)	0.95 ± 0.04	0.81 ± 0.05	0.81 ± 0.04	0.84 ± 0.11	/
LVPWs (mm)	1.55 ± 0.06	1.28 ± 0.06	1.53 ± 0.11	1.27 ± 0.02	¶¶
EDV (mL)	0.098 ± 0.005	0.102 ± 0.006	0.142 ± 0.016 ^$^	0.14 ± 0.014 ^$$^	§§§
ESV (mL)	0.016 ± 0.001	0.024 ± 0.002 *	0.025 ± 0.004 ^$^	0.046 ± 0.006 ^$$$,^***	¶¶¶, §§§, i
LVEF (%)	84.1 ± 0.5	76.3 ± 1.1 *	82.6 ± 1.4	66.7 ± 5 ^$$$,^***	¶¶¶, §§§, i
LVFS (%)	46.9 ± 0.6	39.4 ± 1 **	45.6 ± 1.5	32.2 ± 3.3 ^$$$,^***	¶¶¶, §§§, ii
SV (mL)	0.083 ± 0.005	0.77 ± 0.004	0.117 ± 0.012 ^$$^	0.094 ± 0.013 ^$,^*	§§
LV (mg)	109 ± 6	94 ± 7	98 ± 5	102 ± 14	/
CO (mL/min)	42.0 ± 2.4	38.0 ± 2.1	63.3 ± 6.9 ^$$^	51.1 ± 5.8 ^$,^*	¶, §§§

**Table 2:** Anatomical and echocardiographic parameters 8 weeks after induction of pressure overload in *Sirt1^f/f^* and *Sirt1^ciKO^* mice. BW, Body weight. TL, Tibia length. HW, Heart weight. HW/BW, Heart weight-to-body weight ratio. HW/TL, Heart weight-to-tibia length ratio. LW/BW, Lung weight-to-body weight ratio. LW/TL, Lung weight-to-tibia length ratio. KW/BW, Kidney weight-to-body weight ratio. KW/TL, Kidney weight-to-tibia length ratio. HR, Heart rate. IVSd, Interventricular septal thickness at end-diastole. IVSs, Interventricular septal thickness at end-systole. LVIDd, Left ventricular internal dimension at end-diastole. LVIDs, Left ventricular internal dimension at end-systole. LVWPd, Left ventricular posterior wall thickness at end-diastole. LVWPs, Left ventricular posterior wall thickness at end-systole. EDV, Left ventricular telediastolic volume. ESV, Left ventricular telesystolic volume. LVEF, Left ventricular ejection fraction. LVFS, Left ventricular fractional shortening. SV, Stroke volume. LV, left ventricular mass. CO, Cardiac output. ANOVA: ¶ *p* ≤ 0.05, ¶¶ *p* ≤ 0.01, ¶¶¶ *p* ≤ 0.001 for the genotype effect; § *p* ≤ 0.05, §§ *p* ≤ 0.01, §§§ *p* ≤ 0.001 for the TAC effect; i *p* ≤ 0.05, ii *p* ≤ 0.01 for the interaction effect. Post hoc Newman–Keuls test: * *p* ≤ 0.05, ** *p* ≤ 0.01, *** *p* ≤ 0.001 *Sirt1^f/f^* vs. *Sirt1^ciKO^* (same surgery); ^$^ *p* ≤ 0.05, ^$$^ *p* ≤ 0.01, ^$$$^ *p* ≤ 0.001 Sham vs. TAC (same genotype).

	*Sirt1^f/f^*		*Sirt1^ciKO^*		2 Way ANOVA
	SHAM	TAC	SHAM	TAC	
BW (g)	28.3 ± 0.4	28.5 ± 0.7	28.7 ± 0.5	28.7 ± 0.8	/
TL (mm)	17.6 ±0.1	17.8 ± 0.1	17.6 ± 0.1	18.2 ± 0.1	/
HW (mg)	153.4 ± 4.4	217.7 ± 12.8 ^$^	158.9 ± 4.0	249.6 ± 24.7 ^$$$^	§§§
HW/BW (mg/g)	5.4 ± 0.1	7.6 ± 0.3 ^$$$^	5.5 ± 0.1	8.7 ± 0.7 ^$$$,^*	§§§
HW/TL (mg/mm)	8.7 ± 0.2	12.2 ± 0.7 ^$$^	9.0 ± 0.3	13.7 ± 1.3^$$$^	§§§
LW/BW (mg/g)	5.2 ± 0.1	5.4 ± 0.2	5.1 ± 0.1	6.2 ± 0.9	/
LW/TL (mg/mm)	8.3 ± 0.2	8.7 ± 0.5	8.4 ± 0.2	9.9 ± 1.6	/
KW/BW (mg/g)	12..3 ± 0.3	11.2 ± 0.3 ^$^	12.1 ± 0.2	11.5 ± 0.3	§
KW/TL (mg/mm)	19.8 ± 0.6	17.9 ± 0.6	19.7 ± 0.6	18.2 ± 0.7	§
HR (bpm)	542 ± 19	541 ± 13	514 ± 23	543 ± 14	/
IVSd (mm)	0.72 ± 0.05	0.93 ± 0.07	0.71 ± 0.04	0.83 ± 0.04	§§
IVSs (mm)	1.36 ± 0.05	1.39 ± 0.07	1.34 ± 0.03	1.21 ± 0.04 *	/
LVIDd (mm)	3.79 ± 0.09	4.03 ± 0.17	3.88 ± 0.1	4.14 ± 0.11	§
LVIDs (mm)	2.11 ± 0.08	2.79 ± 0.16 ^$$$^	2.24 ± 0.08	3.19 ± 0.11 ^$$$,^*	¶, §§§
LVPWd (mm)	0.71 ± 0.03	0.81 ± 0.08	0.67 ± 0.03	0.87 ± 0.04^$^	§§
LVPWs (mm)	1.25 ± 0.04	1.23 ± 0.06	1.21 ± 0.04	1.14 ± 0.02	/
EDV (mL)	0.140 ± 0.009	0.152 ± 0.014	0.149 ± 0.011	0.179 ± 0.012	/
ESV (mL)	0.026 ± 0.003	0.061 ± 0.011 ^$$^	0.031 ± 0.003	0.086 ± 0.008 ^$$$,^*	¶, §§§
LVEF (%)	81.7 ± 0.9	65.0 ± 2.2 ^$$$^	79.5 ± 1.1	52.5 ± 2.4 ^$$$,^***	¶¶¶, §§§, ii
LVFS (%)	44.5 ± 1.0	30.8 ± 1.4 ^$$$^	42.4 ± 1.0	23.0 ± 1.3 ^$$$,^***	¶¶¶, §§§, i
SV (mL)	0.11 ± 0.1	0.11 ± 0.01	0.12 ±0.01	0.09 ± 0.01	/
LV (mg)	92 ± 4	136 ± 17 ^$$^	91 ± 5	135 ± 10 ^$$$^	§§§
CO (mL/min)	61.3 ± 3.9	54.3 ± 5.3	60 ± 4.6	50.8 ± 4	/

**Table 3:** **Antibodies.** SIRT1, sirtuin 1. Ac-H1, acetylated histone 1. FoxO1, forkhead box O protein 1. Ac-FoxO1, acetylated forkhead box O protein 1. Ac-p53, acetylated tumor protein 53. CS, citrate synthase. VDAC1, voltage-dependent anion channel 1. ACC, acetyl-CoA carboxylase. pACC, phosphorylated acetyl-CoA carboxylase. AMPK, AMP-activated protein kinase. pAMPK, phosphorylated AMP-activated protein kinase. SOD2, superoxide dismutase 2.

Antibody	Company	Catalog No	Dilution
SIRT1	Abcam	ab110304	1000
Ac-H1	Sigma	H7789	1000
FoxO1	Cell signaling	2880S	250
Ac-FoxO1	Santa Cruz	sc49437	1000
Ac-p53	Cell signaling	2570	250
CS	Abcam	ab96600	1000
VDAC1	Cell signaling	4866	1000
ACC	Cell signaling	3676	1000
pACC	Cell signaling	3661	1000
AMPK	Cell signaling	2532	500
pAMPK	Cell signaling	2531	500
SOD2	Abcam	ab16956	500
Total OXPHOS	MitoSciences	MS604	250
Actin	Santa Cruz	SC-8432	200
Tubulin	Sigma	T6199	1000

## Abbreviations

HF	Heart failure
CVD	Cardiovascular Diseases
SIRT	Sirtuin
PGC-1α	Peroxisome proliferator-activated receptor gamma coactivator 1α
MHC	Myosin heavy chain
TAC	Tranverse aortic constriction
LV	Left ventricle
CS	Citrate synthase
COX	Cytochrome c oxidase
p53	Tumour suppressor p53 protein
H1	Histone H1
FoxO1	Forkhead box protein O1
LVEF	Left ventricular ejection fraction
LVFS	Left ventricular fractional shortening
LVPWs	End-systolic left ventricular posterior wall thickness
LVIDs	End-systolic left ventricular internal diameter
ESV	End-systolic volume
IVSs	End-systolic interventricular spetal thickness
HW	Heart weight
BW	Body weight
TL	Tibia length
AK	Adenylate kinase
CK	Creatine kinase
VDAC	Voltage-dependent anion channel
AMPK	AMP-activated protein kinase
ACC	Acetyl-Coa carboxylase
ROS	Reactive oxygen species
ER	Endoplasmic reticulum
